# Immuno-physiological role of exogenous enzymes supplementation in heat stressed growing calves

**DOI:** 10.1038/s41598-024-78590-1

**Published:** 2024-11-13

**Authors:** Sherif Yousif Eid, Sana Sayed Emara, Ahmed Kamel Sharaf, Omar A. Ahmed-Farid, Hussein Mostafa El-Zaher

**Affiliations:** 1 Biological Applications Department, Radioisotopes Applications Division, Nuclear Research Center, Egyptian Atomic Energy Authority, POB 13759, Cairo, Egypt; 2 Department of Physiology, Egyptian Drug Authority, POB 12553, Giza, Egypt

**Keywords:** Exogenous enzymes (ZADO), Heat stress, Immunoglobulins, Thyroid hormones, Growing calves, Animal physiology, Biochemistry, Climate change

## Abstract

Twenty Crossbred (Baladi× Brown-Swiss) male growing-calves, 6 to 8 months old, weighing an average of 115 to 125 kg were subjected for evaluating ZADO supplementary effects on blood biochemical parameters, antioxidant status, thyroid activity, and growth efficiency of calves under semi-arid conditions. The experimental units were pinned equally in two groups. The control (G1) fed the basal ration, and the 2nd, treatment group (G2) fed the basal ration + a daily supplement of 10 g ZADO (exogenous enzymes) calf^− 1^ day^− 1^ in a powder form mixed well with ration. The results showed that ZADO treatment reduced (*P* < 0.001) each of respiration rate (RR), rectal temperature (RT), serum malondialdehyde (MDA), urea, creatinine, and cortisol. Also, caused a marked decline in oxidized glutathione (GSSG) and alanine aminotransferase (ALT) activities. However, supplemented ZADO to growing calves diets improved antioxidant status including reduced glutathione (GSH) and superoxide dismutase (SOD) activities, total antioxidant capacity, serum total protein, albumin, and globulin, as well as IgG and IgM besides. ZADO enhanced (*P* < 0.01) thyroid activity, and feed efficacy parameters. Finally, supplementing growing calves with ZADO under semi-arid circumstances alleviates the heat stress effect, and leads to an improvement in calves’ growth performance.

**Supplementary Information:**

The online version contains supplementary material available at 10.1038/s41598-024-78590-1.

## Introduction

One of the greatest challenges facing the livestock sector in Egypt is heat stress. It may impair farm animals’ physiological, reproductive, and productive abilities^1,2^ Global warming and climatic changes have become major threats to animal production^3^. As climate change progresses, periods of high temperatures and humidity are likely to become more frequent and intense and may persist for longer durations. Summer temperature in the Mediterranean region, including Egypt, is generally characterized by high ambient temperature and relative humidity (RH), which is outside of cattle- especially crossbred -comfort zone resulting in heat stress^4^.

Heat stress-related biochemical and physiological changes, such as an increase in the production of free radicals and reactive oxygen species, can lead to oxidative stress^5^. Probiotics may play a beneficial role in several health conditions and performances on host biological functions, including gut microflora, intestinal microbial composition and toxicity, metabolic effects, and immunomodulation^6,7^.

Feed additives enclosing exogenous bacteria and yeast are widely used in manipulating ruminal fermentation to boost livestock productivity^8^. The biological additives are live microbial feed supplements that beneficially improve the microbial balance of the host animal. ZADO, a commercial admixture of exogenous enzymes (EZ) derived from anaerobic bacteria rich in cellulolytic enzymes^9,10^. Its main action are on rumen kinetics and feed utilization by ruminal microflora to enhance fermentation and the digestibility of nutrients by enhancing fiber degradation via attachment and/or improve access of the ruminal microorganisms to the cell wall and accelerate the rate of digestion^11,12^. ZADO has been shown to improve feed intake, feed efficiency, and milk production in animals by enhancing the digestibility of dry matter, crude protein, acid detergent fiber, net detergent fiber, and volatile fatty acids concentration in rumen^13,14^. The supplementation of EZ resulted in an increase in the energy utilization of forage, which could potentially impact the immune system indirectly. This is because immune nutrition relies on the proper utilization of dietary resources to support the protection of immunocompetence^15^. Furthermore, studies have indicated that adding biological agents to the diet can positively influence lipid metabolism^16^.

While the immuno-physiological responses to ZADO as a biological additive in heat-stressed crossbred-calves are limited and argumentative, this manuscript aimed to use ZADO to reduce the negative effects of hot weather on the immune system and the vital functions of crossbred calves under hot conditions in Egypt, which in turn leads to raising the productive efficiency of calves.

## Materials and methods

### Ethics approval of animal experiments

The experimental proposal, procedure, and animal treating were approved by Research Ethics Committee (REC-NCRRT; Protocol No.43 A/23) for experimental studies of the Egyptian Atomic Energy Authority which organized and operated according CIOMS&ICALAS International Guiding Principles for Biomedical Research 2012. All methods used in this study are reported in accordance with ARRIVE guidelines.

### Farm geographical coordinates

This experiment was performed at the Cattle Production Project Farm, Nuclear Research Centre, Egyptian Atomic Energy Authority, The farm is in a semi-barren region, site coordinates 30º17’36.6"Nº31 23"34.3” E, belonging to Inshas City, Egypt.

### Animals’ assemblage and experimental scheme

The experimental diets were provided for a two-week adaptation period, and the experimentation lasted for six weeks after. Twenty male crossbred calves (Baladi (B) x Brown Swiss (BS), 6 to 8 months old, weighing an average of 115 to 125 kg, were used. The animals were randomly pinned equally in two groups (10/group). The control (G1) offered the basal diet, and the treatment (G2) received a control diet + 10 g ZADO /calf/day (According to manufacturer’s recommendation dosage for ruminants) in a powder form mixed well with ration. ZADO is a commercial exogenous enzyme mixture made from anaerobic probiotic bacteria; each 1 g of ZADO powder contains *Ruminococcus sp*. 28 × 10 ^4^ CFU. The mixture was prepared by BACTIZAD Company for feed additives manufacturing, Belbase, El-Sharkya Governorate, Egypt.

### The ration and rational regime

The diet quotas were obtainable for the groups at 10 a.m. daily. The concentrate of feed mix (CFM) and rice hay were given based on the animals’ live body weight averages, as stated by NRC^17^ recommendations. Fresh, clean tap water was freely accessible for all animals.

Samples of concentrate rations were biweekly collected for lab analyses. The chemical compositions and feedstuffs’ nutritional values are estimated in the central lab for soil, food, and feedstuff (CLSFF) according to AOAC^18^ the laboratory accredited with ISO 17,025/2017, as in Table 1. The percentage of CFM ingredients were; 35% yellow corn, 44.5% wheat bran, 15% undecorticated cotton seed meal, 1% molasses, 2% Glutophide, 1% lime stone, 1% sodium chloride, 0.2% minerals mixture (each Kg contains: 881.6 g CaCo3, 30 g Fe, 45 g Zn, 40 g Mn, 5 g Cu, 0.3 g I, and 0.1 g Se ), 0.1% vitamin mix (each Kg includes: 2 million (IU) vit D3, 20 million (IU) vit A, and 2 g vit E), 0.1% sodium bicarbonate, and 0.1% antitoxin.

**Table 1:** Feedstuffs’ chemical analysis and nutritional values based on dry matter. % NFE = % DM - (% EE +% CF+ % CP + % ash ) *, GE (Mcal/kg DM) = 0.057 CP % + 0.094 ether extract (EE) % + 0.0415 carbohydrate **, NE (Mcal/kg DM) = 0.0245 X TDN % − 0.12 (NRC, 2001);*** TDN (%/kg DM) according to the Central Lab for soil, Food and Feedstuff (CLSFF), Faculty of Technology and Development, Zagazig University, Accredited according to ISO 17,025/2017.

	Chemical composition								Nutritive values		
	Moisture	DM	OM	CP	CF	EE	Ash	NFE	GE (Mcal/kg DM)*	NE (Mcal/kg DM)**	TDN (%/kg DM)**
CFM	7.96	100	86.73	13.7	8.64	4.8	13	59.6	4.06	1.47	65
Rice- Hay	7.5	100	81.82	3.2	34.1	1.9	18	42.6	3.55	0.94	43.24

### Animal management

Each group served well in one yard. Throughout the whole experiment, the calves were settled in three distinct (40 × 40 m) dirt floor yards that were encircled by a 1.5-metre-high pipe fence. The middle one-third yard is a shaded-roof area (3.5 m height) with natural ventilation. Each yard is provided with troughs and a source of drinking water to always be available automatically.

### Meteorological data

Throughout the trial, the animals were maintained in the same environmental states in a shaded free-stall barn. The experiment was carried out during July and August 2023, for two months. Air temperature (*Ta*) and the relative humidity (RH) were daily recorded using a thermo-hygrometer data logger of the EAEA meteorological station at 7am and 3pm throughout the trail, and their means were computed, where the *Ta* and the RH at 3pm hrs were averaged 36.24 °C ± 0.33 and 59.48% ± 0.74, respectively (THI = 88.4). At 7am hrs. *Ta* average was 27.80 °C ± 0.24 and RH was 77.23% ± 0.71, (THI 79). THI calculation equation was THI = (0.8×*Ta* °C) + [(RH %)× (*Ta* °C -14.4)/100)] + 46.4^19^, . Knowing that THI limits for cattle are: comfort (THI < 68), mild-discomfort (68 < THI < 72), discomfort (72 < THI < 75), alert (75 < THI < 79), danger or severe heat stress (79 < THI < 84), and emergency or very severe heat stress (THI > 84).

### Blood sampling and estimated parameters

Blood sampling was done prior to feeding from the neck main vein at the launch and end of the experimental period. The collected samples were placed in an ice box right away to the lab. Clear serum was extracted from clotted blood using centrifugation at 3000 × for 20 min, and it was kept at -20 °C for subsequent analyses. The following parameters and biomarkers were estimated using colorimetric spectrophotometer technique of the commercial kits manufactured by Spin React, S.A./S.A.U. Ctra. Santa. Coloma, Spain, unless otherwise indicated: total protein (TP) albumin, aspartate aminotransferase (AST), alanine aminotransferase (ALT), urea, and creatinine. The colorimetric method was employed to estimate the serum total antioxidant capacity (TAC), malondialdehyde (MDA), as well as the antioxidant enzyme activities in terms of oxidized glutathione (GSSG), superoxide dismutase (SOD), and reduced glutathione (GSH) using the commercial kits manufactured by Diamond Diagnostic Company, Giza, Egypt. The Eagle Biosciences Cortisol ELISA Assay Kit (Catalog Number: COR31-K01 (1 × 96 wells)) was used for the quantitative measurement of serum cortisol by an enzyme immunoassay. Serum immunoglobulins were estimated via double antibody sandwich ELISA kits for Bovine IgG and IgM; Catalog Numbers: BGG69-K01 and BCM61-K01 (1 × 96 wells), with detection limits of 0.1–100 ng/mL and 0.05-50 ng/mL, respectively. Eagle Biosciences, Inc. 20 A NW Blvd, Suite 112, Nashua, NH 03063 USA. Serum concentrations of triiodothyronine (T_3_), and thyroxine (T4) were determined by using ^125^ I-RIA antibody-coated tubes kits purchased from Dia-Source ImmunoAssays S.A. Rue du Bosquet, 2, B-1348 Louvain-la-Neuve (Belgium).

### Physiological parameters and growth performance

Respiration rate (RR) & rectal temperature (RT) as a physiological response to heat stress were measured before feeding three times per week. A clinical thermometer was inserted into the rectum for two minutes to get RT, while the count of the flanks movement for one minute (complete inward and outward movement of the flank was counted as one respiration) using a stop watch for expressing RR as breath/min. The RR measurement was carried out before measuring the rectal temperature to avoid animal excitation. Calves’ body weighing was assessed prior to daily nutrition and watering at the start and end of the trail. Growth indicators such calf’s daily body weight and total gains were calculated; also, DMI and Gain/feed were computed and expressed as kg /day^−^/calf and kg gain/ kg DMI, respectively. Body weight gain was calculated by subtracting the average initial live body weight of each animal from the average final body weight for the same animal. Dry matter intake (DMI) was determined by calculating the average of intake kg/calf/day over the course of experiment by recording the residual for each group 3 times a week. Gain/feed was calculated as kg gain/kg DMI and showed as the average in the results.

### Statistical analysis

SAS^20^ (Statistical Analysis System software version 2009) was used to test the significance of the differences between the mean values of the treatment and control groups using the unpaired “t” test. According to the model: Yij = µ + Ti + eij; Where, Y = the dependent variable, µ = overall mean, Ti = the fixed effect of treatment (1 = control, 2 = treatment), eij = random error.

## Results

### Implications of supplementary exogenous enzymes (ZADO) on heat-stressed growing calves

1- Physiological responses

Table 2 illustrates the physiological measurements of heat-stressed calves versus ZADO treatment group. Both RT and RR of treatment were lower (39.35 °C and 50.6 breath/min; *P* < 0.05) than control with change rate − 1.3% and − 29.53%, respectively due to ZADO treatment.

**Table 2:** Physiological measurements of heat-stressed calves supplemented exogenous enzymes ZADO. RT rectal temperature, RR respiration rate. Data presented as mean ± SE. P-value indicates the significant deference between means in the same raw.

Variables	Groups		*P*- value	Change %
	Cotrol	ZADO		
RT (°C)	39.87 ± 0.037	39.35 ± 0.05	0.05	-1.3
RR (Breath/min)	71.80 ± 0.95	50.60 ± 0.68	0.05	-29.53

2- Oxidant and antioxidant status

**Fig. 1:**
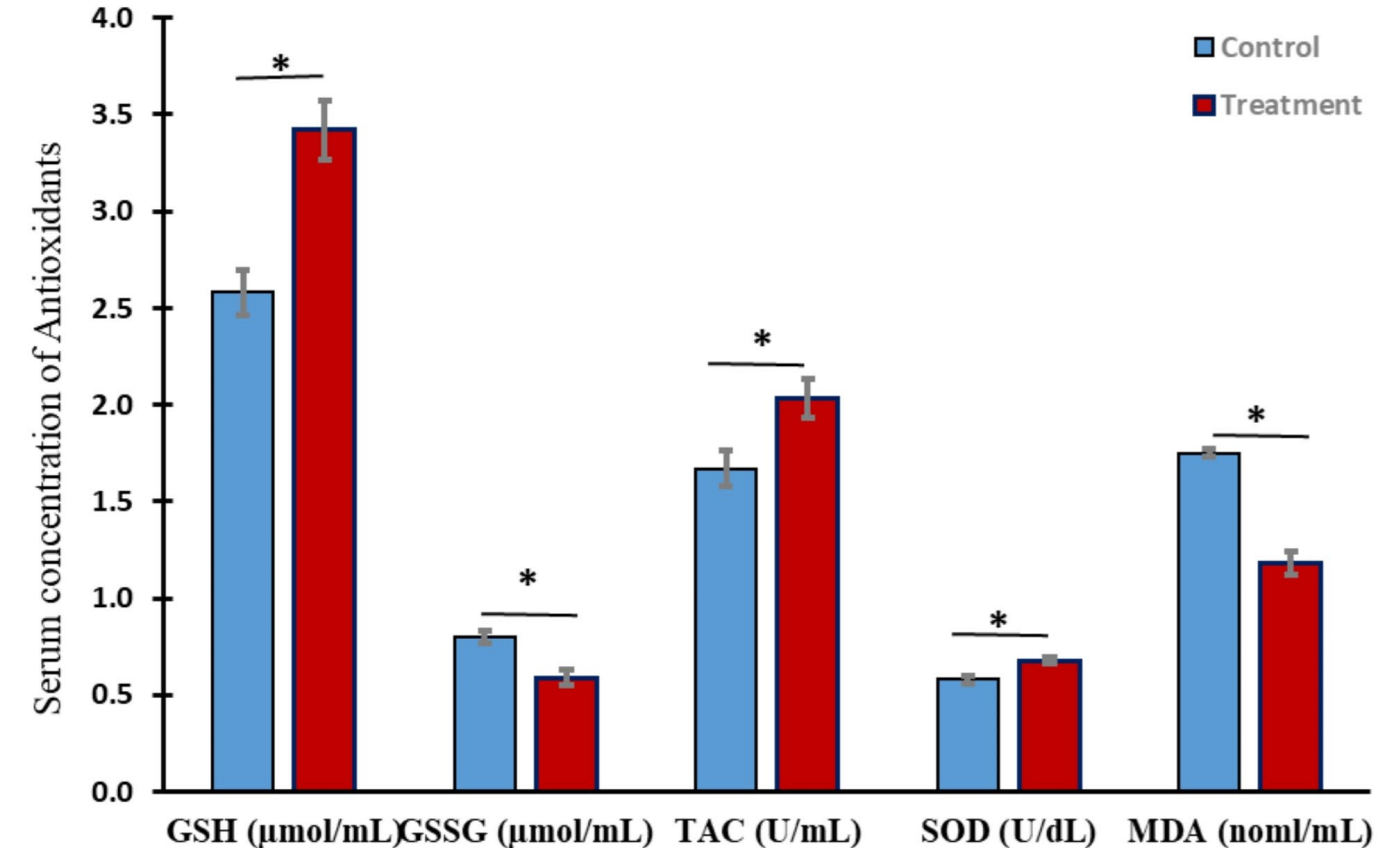
ZADO effects on serum antioxidants activities of heat stressed calves. GSH reduced glutathione, GSSG oxidized glutathione, TAC total antioxidant capacity, SOD superoxide dismutase, MDA malondialdehyde. Data displayed as mean ± SE. Asterisk indicates the difference (*P* < 0.01) between means.

Figure 1 presents antioxidant parameters of heat-stressed growing calves that supplemented ZADO. ZADO treatment significantly(*P* < 0.01) improved antioxidant efficiency throughout increasing GSH, TAC, and SOD activities than the control group with change rate values of 32.24%, 21.57%, and 16.7%, respectively. Treatment also decreased (*P* < 0.01) GSSG and MDA by -26.09 and 32.95% less than control.

3- Blood proteins and biochemical parameters

**Fig. 2:**
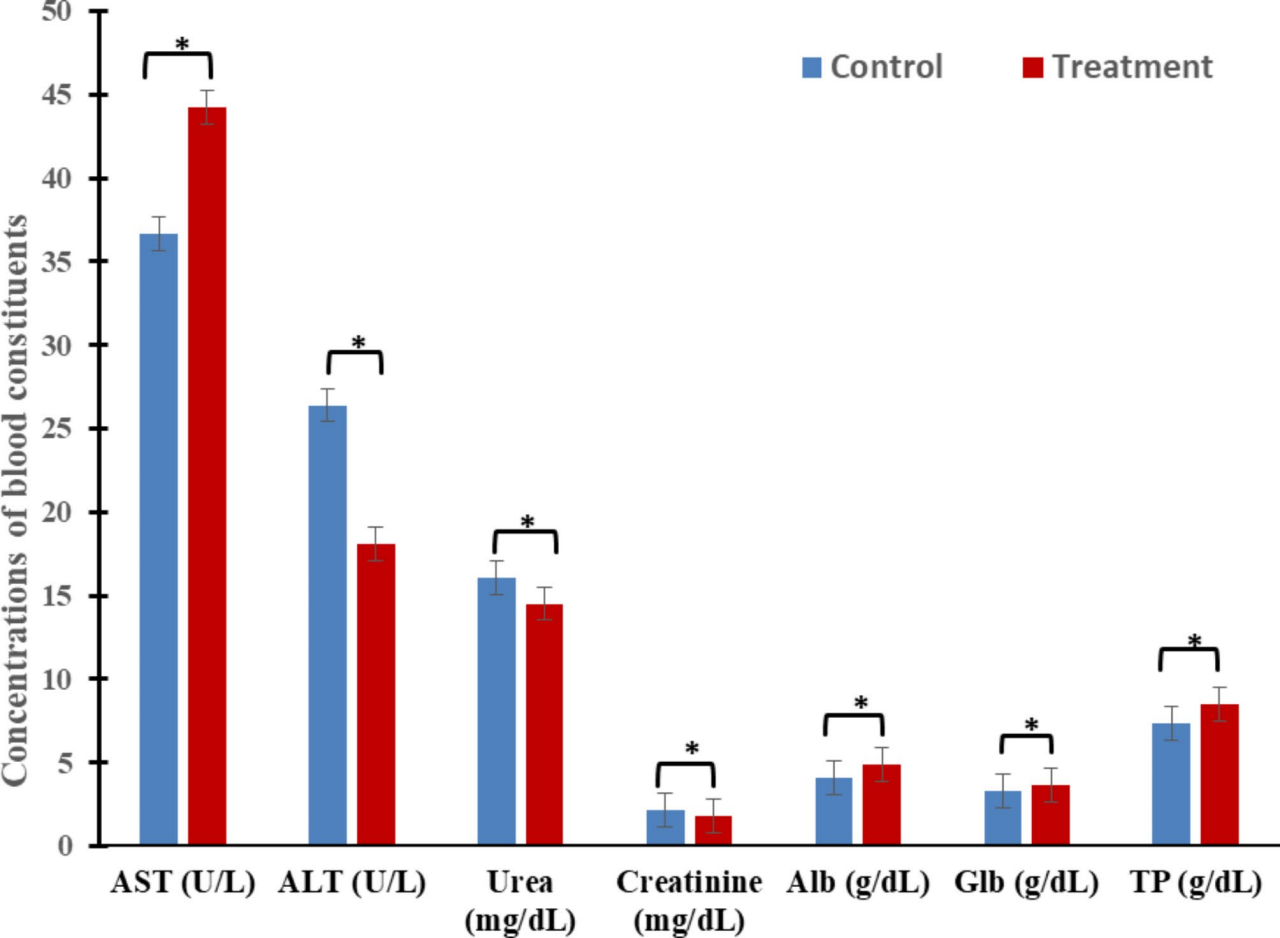
Exogenous enzymes ZADO effects on the liver, kidney function, and blood biochemical (means ± SE) of heat-stressed growing calves. AST Aspartate aminotransferase, ALT Alanine aminotransferase, Alb Albumin, Glb Globulin, TP Total protein. Data displayed as mean ± SE. Asterisk indicates the difference (*P* < 0.05) between means.

A marked increase (*P* < 0.01) in serum AST was recorded for the treated group as compared to the control with a positive change rate of 20.81% (Fig. 2). Whereas, ALT, urea, and creatinine were significantly lower by -31.59%, -9.45%, and − 17.0%, respectively than control, as in Fig. 2. In addition, ZADO increased (*P* < 0.01) serum Alb, Glb, and TP in comparison to control with positive change rates of 20.81%, 19.86%, 10.94% and 15.87%, respectively (Fig. 2).

4- Immunity and hormones

**Fig. 3:**
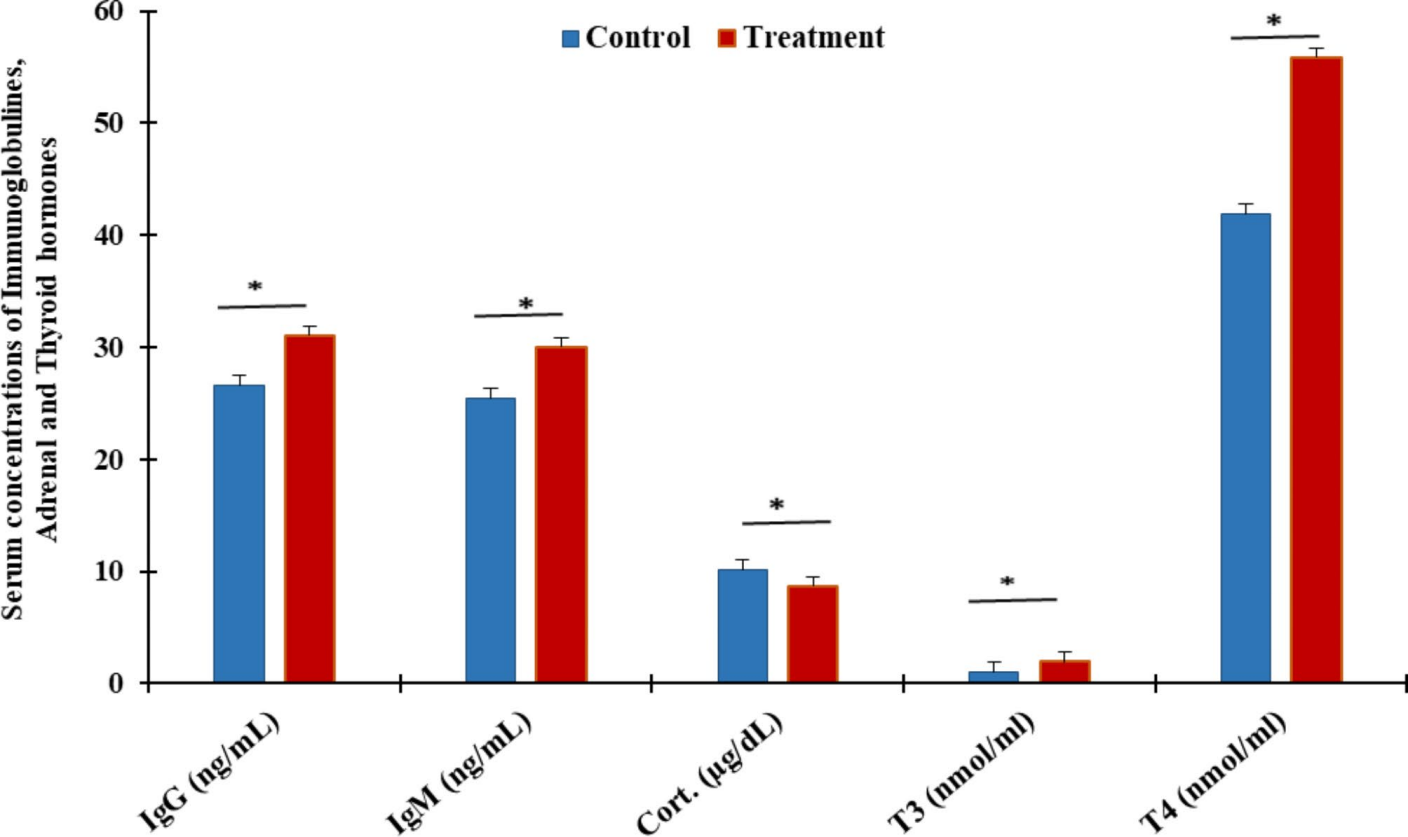
Immunoglobulins, adrenal and thyroid gland hormones as affected by exogenous enzymes ZADO treatment in heat-stressed growing calves. IgG Immunoglobulin G, IgM immunoglobulin M, Cort cortisol, T3 Triiodothyronine, T4 Thyroxin. Data penned as mean ± SE. Asterisk indicates the difference (*P* < 0.01) between means.

Supplementation of ZADO increased (*P* < 0.01) IgG and IgM levels over control by 16 and 18%, respectively as shown in Fig. (3). Furthermore, ZADO treatment enhanced thyroid gland activity via increasing serum T3 and T4 levels more than control with change values of about 81.67 and 33.49%,respectively. On the other side, ZADO calves had a -13.96% decrement (*P* < 0.01) in cortisol hormone less than control (Fig. 3).

5- Growth performance

Table 3 illustrates the growth performance of heat-stressed calves versus ZADO treatment group. There is no significant (*P* = 0.07) difference in IBW (Kg) between the two groups but at the end of the experiment, ZADO calves recorded 162.89 Kg, *P* < 0.001 FBW higher than the control with a change rate value 7.75%. Supplementation of heat-stressed calves with ZADO increased (*P* < 0.001) DFI, DG, and subsequently TG more than the control group with change rates of 7.05%, 31.51%, and 32.72%, respectively. In addition to a significant rise (0.22 kg/kg; *P* < 0.001) in G/F ratio over control by the rate of 27.78% for ZADO group.

**Table 3:** Growth performance traits of heat-stressed calves supplemented ZADO exogenous enzymes. IBW initial body weight, FBW final body weight, TG total gain, DG Daily gain, DFI Daily feed intake, G/F gain/feed ratio. Data expressed as mean ± SE. P-value indicates the significant deference between means in the same raw.

Variables	Groups		*P*-Value	Change %
	Control	Treatment		
IBW (Kg)	118.49 ± 0.42	119.51 ± 0.33	0.07	0.86
FBW (Kg)	151.18 ± 0.51	162.89 ± 0.45	0.001	7.75
TG (Kg)	32.69 ± 0.52	43.38 ± 0.22	0.001	32.72
DG (Kg/d)	0.73 ± 0.01	0.97 ± 0.01	0.001	31.51
DFI (Kg)	3.97 ± 0.037	4.25 ± 0.04	0.001	7.05
G/F ratio (Kg/Kg)	0.18 ± 0.003	0.22 ± 0.002	0.001	27.78

## Discussion

Seasonal variations, particularly hot summer temperatures in Egypt, have substantial impacts on growing calves’ physiological variables (i.e., RT & RR)^21^. In accordance with current findings and explanations Gado et al.^22^ demonstrated that exogenous enzyme addition to the feed improved normal animals’ vital signs (e.g., R.R., skin temperature, and R.T). This indicates the enhancement of body thermoregulation with the consumption of the exogenous enzyme ZADO via the regulation of body metabolism and respiratory rate.

Heat stress substantially contributes to oxidative stress in mammals^23^, resulting in increased MDA levels in cattle exposed to elevated temperatures compared to those in a thermoneutral condition^24^. In terms of the correlation between high summer temperatures and oxidative stress, Purwar et al.^25^ suggested that incorporating feed additives into the animals’ diet could mitigate the adverse effects of oxidative stress induced by heat stress in hot summer conditions. It is worth mentioning that ZADO considerably (*P* < 0.01) increased GSH, TAC, and SOD while decreasing GSSG and MDA (Fig. 1). In broiler chickens, GSH, SOD, and MDA showed a similar pattern in response to the inclusion of fibrolytic enzymes in the feedstuff^26^. Likewise, Castillo et al.^27^ demonstrated that probiotics significantly decreased MDA activity in serum and improved calf health.

Under hot conditions, both AST and ALT enzyme activities elevate greater than winter in cows^28^ due to the increased gluconeogenesis process by corticoids^29^. The present results of blood serum metabolites parameters, which recorded an increase in AST and a decrease in ALT activity due to exogenous enzymes ZADO supplementation (Fig. 2) disagree with Gado et al.^30^ who found no effects for ZADO on AST and ALT activities. Also, the minor effects of the additive on AST and ALT activities, within the normal physiological range^31^, indicate that additives did not affect liver function^32^. The results of AST activity are in opposition to those of Guo et al.^33^, who reported that probiotics significantly decreased AST activity in serum. The decrease in ALT activity from our results led to the conclusion that ZADO may improve liver health. Numerous studies have demonstrated that urea-N was greatly higher (*P* < 0.05) under summer season than the winter in Holstein cows, crossbred and Baladi cows, and growing heat-stressed calves **[28**,** 34**,** 35**, respectively**].** Salles et al.^36^ also observed an increase in serum urea-N and creatinine of heat-stressed animals. According to Erasmus et al.^37^ and Kamiya et al.^38^, there could be two possible causes of the increase in urea-N throughout heat stress times: enhanced rumen nitrogen balance or increased muscle breakdown. Likewise, the low energy/protein ratio and gluconeogenesis process might be the cause of the high level of urea-N insufficient energy conditions for growth^39^.

The results of blood serum metabolites parameters, which recorded a decrease in urea and creatinine concentration due to ZADO (Fig. 2), partly agree with Ashour et al.^40^ results on camels, who found a decrease (*P* > 0.05) in blood urea and creatinine in ZADO treatment group, pointing to an enhancement in the nutritional status and kidney function without any impairment in protein catabolism^41^. Simultaneously, a notable reduction in TP concentration, Alb, and globulin has been observed during the times of heat stress^42^. Helal et al.^43^ attributed this reduction to the vasoconstriction and a decrease in plasma volume in heat-stressed goats. The increment in blood proteins obtained in the current study because of ZADO treatment (Fig. 2) matches those in camels^40^ and weaned calves^44^. The increase in blood proteins for G1 may be due to the superior utilization of feed protein and ruminal true protein-N across the digestive passage^45^. This indicates that ZADO enhances the flora and fermentation processes in rumen, which leads to the greatest diet’s utilization. On the other hand, exogenous fibrolytic enzymes (EFE) additives increase the Alb and Glb, suggesting improved nutrient utilization without impairing protein catabolism in muscles^41^.

Immunoglobulins, which are mostly found in serum, are crucial for reacting with antigens and boosting the antiviral and antibacterial capacities of weaned animals. IgG is the key antibody constituent in the blood with significant immunological effects. IgM acts as the first defense border against infections and plays a valuable role in immunological regulation and tolerance. The increase in immunological parameters in the present study agrees with those of Rao et al.^46^, who implied that exogenous enzymes supplemented to feed diets improve immunological traits such the increase of IgA, IgM, and IgG immunoglobulins^47^. Probiotics administration aims to modulate the host immune response against potentially harmful antigens via increasing plasma immunoglobulins level, activation of lymphocytes, and antibodies’ production^48,49^. Globulins are a main proteins in the blood, therefore, a raise in globulin may represent an increase in the ability to produce additional IgG and IgM. Ercal et al.^50^ found that elevated oxidative stress results in reduced levels of immunoglobulins and antioxidant enzymes, and consumption of fat which may modify immunoglobulin levels (IgG and IgM). For immune response modulation, probiotics through pattern recognition receptors, i.e. Toll-like receptors, on the immune cells could regulate vital signaling pathways, producing nuclear factor kb and nitrogen-activated protein kinases, and communicate with the host^51^ to activate or stimulate innate immunity which resulting in pre and anti-inflammatory cytokines or chemokines’ production^52^. From another side, Probiotics act to improve intestinal microbiota balance by stimulating the growth/activity of beneficial bacteria and suppressing those of harmful bacteria. Although the mechanism of probiotics on gut microbiota modulation remains unclear, probiotics could restrain the growth of pathogens through the production of short-chain fatty acids (SCFA) and toxins^53^ and the competition of colonization sites with pathogens^54^.

The exposure to heat stress decreases plasma T3 and T4 levels in cows, which may help the animals to reduce their internal heat production, consequently diminish heat gain^28,55^. The obtained results of thyroid activity, which recorded an increase in thyroid gland activity via T3 and T4 due to exogenous enzymes ZADO (Fig. 3) agree with Hamdon et al.^56^ who found that ZADO significantly increased the concentrations of T3 and T4 in growing lambs, as well as due to probiotic usage^57^. Probiotics potentially affect the thyrotropin-releasing hormone in the hypothalamus and enhance thyroid gland activity, resulting in an increase in T3 and T4 release in the bloodstream, which together regulate energy consumption, and these are basic in controlling body heat, weight, muscle potency, and the neurological function, as well as, lipids metabolism, lipoprotein balance, and genes of glycolysis and gluconeogenesis^58^. Cortisol is crucially involved in various physiological functions, especially energy production and thermal regulation^59^. Its levels significantly increase under high ambient temperatures of heat stress^60^ as a result of activation of the hypothalamic–pituitary–adrenal (HPA) axis^61^. From our results ZADO treatment had a decrement in cortisol hormone (Fig. 3), this result goes in harmony with those of Naglaa and Ghada^62^, who reported that ewes had lower serum cortisol concentrations after being fed probiotics for 60 days. In addition, heat-stressed dairy cattle were more feed efficient when fed probiotics^63^. Likewise, probiotics supplementation restored cortisol levels to physiological limits in response to heat stress^64,65^. This lead to suggest that probiotics may normalize the activity of the HPA axis and correcting dysfunction of the HPA axis induced by stress.

Heat-stressed calves show a depression in growth performance, especially daily gain^35^; this may be attributed to the undesirable changes in protein metabolism, blood constituents, and metabolic hormone^66^. The current results showed a greater increase in FBW, TG, DG, DFI, and G/F of growing calves exposed to heat stress by using ZADO exogenous enzyme than the control group (Table 3). These are in harmony with the finding of El-Kady et al.^67^, Malik and Bandla^68^, , and Thakur et al.^69^, who recorded a significant increase in average daily gain, total body weight gain, feed conversion as (kg DM/kg gain) in the treated animals groups with ZADO exogenous enzyme. Feeding ruminants with exogenous enzymes has a beneficial impact on diet utilization, animal growth, and productivity^12^. This phenomenon implies a potential enhancement in the overall microbial population within the rumen and the synthesis of microbial proteins^70^. Fibrolytic enzymes inclusion in the diet can boost feed conversion and weight gain in steers^71^ or calves via adjusting nutrient digestibility^72^. Likewise, Wang et al.^73^ observed a similar trend: after weaning, rumen microbial enzyme activities, such as carboxymethyl, cellulase, cellobiase, xylanase, and pectinase, were higher in calves supplemented with exogenous enzymes than in calves not supplemented. The numerous polysaccharidase and xylanase enzymes in exogenous fibrolytic enzymes break down linkages in cellulose and hemicellulose, freeing soluble saccharides and offering vital nutrients or growth factors for rumen microorganisms, consequently enhancing feed utilization and growth patterns^44,74–76^. Conclusion, Supplementing growing calves with 10 g/calf/day ZADO under Egyptian hot summer environmental conditions positively affected growth potentials, body physio-immunological estimated parameters, serum metabolites, and antioxidant capacities.

## Electronic supplementary material

Below is the link to the electronic supplementary material.

Supplementary Material 1

## Data Availability

Data is provided within the supplementary information files.
