# Supplementary material for: Immuno-physiological role of exogenous enzymes supplementation in heat stressed growing calves
Source: Sci Rep. 2024 Nov 13;14:27865. doi: 10.1038/s41598-024-78590-1 (PMC11561112; doi:10.1038/s41598-024-78590-1)
Supplement: Supplementary file 1 — Supplementary Material 1 [file 41598_2024_78590_MOESM1_ESM.doc]

**Table 1. Serum antioxidants variables as affected by exogenous enzymes ZADO® supplementation in heat stressed growing calves.**

| Variables | groups | | *P*-value | Change % |
| --- | --- | --- | --- | --- |
|  | Control | Treatment |
| GSH (µmol/mL) | 2.58±0.12 | 3.42±0.15 | 0.001 | 32.29 |
| GSSG (µmol/mL) | 0.80±0.03 | 0.59±0.04 | 0.001 | -26.09 |
| TAC (U/mL) | 1.67±0.09 | 2.03±0.10 | 0.016 | 21.57 |
| SOD (U/mL) | 58.02±2.09 | 67.72±1.68 | 0.002 | 16.71 |
| MDA (noml/mL) | 1.75±0.02 | 1.18±0.06 | 0.001 | -32.95 |

GSH reduced glutathione, GSSG oxidized, TAC total antioxidant capacity, SOD superoxide dismutase, MDA malondialdehyde Data presented as mean±SE. P-value indicates the significant deference between means in the same raw.

**Table 2. Exogenous enzymes** ZADO® treatment effects on the liver, kidney function, and blood biochemical (means±SE) of heat-stressed growing calves.

| Variables | groups | | *P*-value | Change % |
| --- | --- | --- | --- | --- |
|  | Control | Treatment |
| AST (U/L) | 36.64±0.79 | 44.26±0.55 | 0.001 | 20.81 |
| ALT (U/L) | 26.40±1.14 | 18.06±0.56 | 0.001 | -31.59 |
| Urea (mg/dL) | 16.03±0.16 | 14.51±0.25 | 0.001 | -9.45 |
| Creatinine (mg/dL) | 2.18±0.03 | 1.81±0.03 | 0.001 | -17.01 |
| Albumin (g/dL) | 4.06±0.08 | 4.87±0.17 | 0.001 | 19.86 |
| Globulin (g/dL) | 3.29±0.05 | 3.65±0.11 | 0.008 | 10.94 |
| TP (g/dL) | 7.35±0.09 | 8.52±0.14 | 0.001 | 15.87 |

AST Aspartate transaminase, ALT Alanine transaminase, Alb Albumin, Glb Globulin, TP Total proteins. Data presented as mean±SE. P-value indicates the significant deference between means in the same raw.

**Table 3. Immunoglobulins, adrenal and thyroid gland hormones as affected by exogenous enzymes** ZADO® treatment in heat-stressed growing calves.

| Variables | groups | | *P*-value | Change % |
| --- | --- | --- | --- | --- |
|  | Control | Treatment |
| IgG (ng/mL) | 26.55±0.34 | 31.04±1.22 | 0.002 | 16.92 |
| IgM (ng/mL) | 25.39±0.39 | 30.04±0.57 | 0.001 | 18.34 |
| Cortisol (µg/dl) | 10.07±0.17 | 8.66±0.15 | 0.001 | -13.96 |
| T3 (nmol/ml) | 1.07±0.03 | 1.95±0.04 | 0.001 | 81.67 |
| T4 (nmol/ml) | 41.87±1.78 | 55.89±1.58 | 0.001 | 33.49 |

IgG Immunoglobulin G, IgM immunoglobulin M, T3 Triiodothyronine, T4 Thyroxin. . Data presented as mean±SE. P-value indicates the significant deference between means in the same raw.
